# Analysis of factors associated with admission to the intensive care unit of children and adolescents with COVID-19: application of a multilevel model

**DOI:** 10.62675/2965-2774.20240068-en

**Published:** 2024-07-01

**Authors:** Lecidamia Cristina Leite Damascena, Aline Roseane Queiroz de Paiva Faria, Nyellisonn Nando Nóbrega de Lucena, Ana Hermínia Andrade e Silva, Talita Tavares Alves de Almeida, Diana de Fátima Alves Pinto, Hemílio Fernandes Campos Coêlho, Ana Maria Gondim Valença

**Affiliations:** 1 Postgraduate Program in Decision Models and Health Centro de Ciências Exatas e da Natureza Universidade Federal da Paraíba João Pessoa PB Brazil Postgraduate Program in Decision Models and Health, Centro de Ciências Exatas e da Natureza, Universidade Federal da Paraíba - João Pessoa (PB), Brazil.; 2 Secretaria de Saúde do Estado da Paraíba João Pessoa PB Brazil Secretaria de Saúde do Estado da Paraíba - João Pessoa (PB), Brazil.

**Keywords:** COVID-19, Coronavirus infections, Child, Adolescent, Logistic models, Multilevel analysis, Social vulnerability, Pediatric intensive care units

## Abstract

**Objective:**

To identify factors associated with hospitalization in the intensive care unit in children and adolescents with COVID-19.

**Methods:**

This was a retrospective cohort study using secondary data of hospitalized children and adolescents (zero to 18 years old) with COVID-19 reported in Paraíba from April 2020 to July 2021, totaling 486 records. Descriptive analysis, logistic regression and multilevel regression were performed, utilizing a significance level of 5%.

**Results:**

According to logistic regression without hierarchical levels, there was an increased chance of admission to the intensive care unit for male patients (OR = 1.98; 95%CI 1.18 - 3.32), patients with respiratory distress (OR = 2.43; 95%CI 1.29 - 4.56), patients with dyspnea (OR = 3.57; 95%CI 1.77 - 7.18) and patients living in large cities (OR = 2.70; 95%CI 1.07 - 6.77). The likelihood of requiring intensive care was observed to decrease with increasing age (OR = 0.94; 95%CI = 0.90 - 0.97), the presence of cough (OR = 0.32; 95%CI 0.18 - 0.59) or fever (OR = 0.42; 95%CI 0.23 - 0.74) and increasing Gini index (OR = 0.003; 95%CI 0.000 - 0.243). According to the multilevel analysis, the odds of admission to the intensive care unit increased in male patients (OR = 1.70; 95%CI = 1.68-1.71) and with increasing population size of the municipality per 100,000 inhabitants (OR = 1.01; 95%CI 1.01-1.03); additionally, the odds of admission to the intensive care unit decreased for mixed-race *versus* non-brown-skinned patients (OR = 0.981; 95%CI 0.97 - 0.99) and increasing Gini index (OR = 0.02; 95%CI 0.02 - 0.02).

**Conclusion:**

The effects of patient characteristics and social context on the need for intensive care in children and adolescents with SARS-CoV-2 infection were better estimated with the inclusion of a multilevel regression model.

## INTRODUCTION

Coronavirus disease 2019 (COVID-19) has caused unimaginable consequences for public health and has led to the loss of thousands of lives.^(1)^ Several countries have mobilized to find strategies to control and combat COVID-19, which has become a public health emergency.

In low- and middle-income countries, the incidence of COVID-19 may be influenced by the social vulnerability of some disadvantaged classes. Vulnerable populations have specific characteristics and behaviors related to greater exposure to the virus, including increased susceptibility to infection, stronger associations between comorbidities and unfavorable outcomes and inequality in access to health care.^(2)^

Coronavirus disease 2019 is typically less severe in children and adolescents.^(3)^ However, these patients were affected by and experienced direct consequences of isolation. The pandemic was associated with profound educational, social and psychological changes and food insecurity, increasing the risk of serious adverse outcomes that may cause more deaths of children and adolescents in the most deprived regions.^(4)^

Therefore, it is important to investigate whether there are determinants of social vulnerability at the individual and contextual levels that lead to unfavorable outcomes for children and adolescents with COVID-19. The objective of this study was to identify factors associated with hospitalization in the intensive care unit (ICU) of children and adolescents with COVID-19.

## METHODS

This was a retrospective, exploratory cohort study that used a quantitative approach to identify whether there are factors associated with ICU admission in children and adolescents with COVID-19 in Paraíba. Paraíba is a state in the Northeast Region of Brazil and has an estimated population of 4,039,277 inhabitants, a population density of 66.70 inhabitants/km^2^, a Human Development Index (HDI) of 0.658 and a Gini index of 0.559. The infant mortality rate is 13.29 deaths per thousand live births.^(5)^

The study population consisted of children and adolescents aged zero to 18 years who presented with severe acute respiratory syndrome (SARS), were hospitalized and had a final diagnosis of COVID-19 between April 2020 and July 2021. The database was made available by the *Secretaria de Saúde do Estado da Paraíba* (SES-PB) containing data from April 2020 to July 2021.

The individual variables referring to sociodemographic data, signs and symptoms were extracted from the spreadsheet made available by the SES-PB, and the contextual variables were obtained through electronic websites.^(5-8)^ Several variables, such as population size, which was divided into small (fewer than 10,000 inhabitants), medium (between 10,000 and 50,000 inhabitants) and large (more than 50,000 inhabitants);^(5)^ the Social Vulnerability Index;^(7)^ and the Municipal Human Development Index^(8)^ underwent discretization. Other numerical variables, including age, population density, Gini index, Family Health Strategy coverage, total number of pediatric beds, health facilities, infant mortality, sewage system and urban road paving, were categorized according to the interquartile distribution.

To determine the optimal use of each variable, all the variables were tested categorically and numerically in the two statistical models; however, some variables did not remain in the fitted models.

Descriptive analysis was performed first, followed by data modeling with a logistic regression model crossing each covariate with the event of interest. The associations between the explanatory variables and the outcome with p values ≤ 0.20 were included in the multivariate model. The *stepwise* technique was performed to obtain the final adjusted logistic regression model, using a significance level of 5%.^(9)^ The receiver operating characteristic (ROC) curve was used to evaluate the quality of the final fitted model.

A multilevel logistic regression model was then developed using a significance level of 5%. In the multilevel modeling, two hierarchical levels were considered: the individuals reported with COVID-19 (level 1) and the municipalities that made the notifications (level 2). The multilevel analysis aimed to separate the effects within each municipality (characteristics of the children and adolescents related to the chance of ICU admission) from the effects between the clusters (characteristics of the municipalities that may be associated with the outcome), considering the same outcome of logistic regression without hierarchical levels.

The initial steps for the determination of the multilevel regression model included centralization of the predictor variables and execution of an empty model, i.e., without explanatory variables, to determine whether the likelihood of ICU admission for children and adolescents with COVID-19 differed among the reporting municipalities. The intraclass correlation coefficient (ICC) was used to quantify the homogeneity of the results between the clusters, representing the proportion of variation between the municipalities. The ICC was calculated as the ratio between the variance in waste at the municipal level and the sum of the variances at the municipal and individual levels. Subsequently, the models were tested with the explanatory variables. First, the associations that were significant in the logistic regression analysis were entered into the multilevel model; however, other variables were tested to obtain the best model, considering a significance level of 5%. The deviance value for choosing the best model and the ROC curve were evaluated to analyze the goodness of fit of the model. To perform the statistical analyses, the free software R, version 4.1.1, was used.^(10)^

This study received the consent of the SES-PB and was approved by the Ethics Committee of the *Centro de Ciências da Saúde*, *Universidade Federal da Paraíba* under the respective CAAE (39914320.2.0000.5188).

## RESULTS

**Figure 1:**
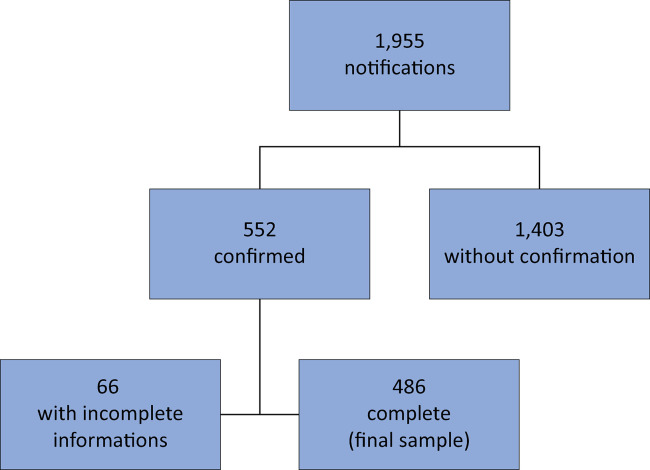
Data screening flow.

Of the 1,955 SARS reports, 552 had a final confirmed diagnosis of COVID-19. Of these, 66 records with multiple missing data points were excluded, resulting in a sample of 486 patients, as shown in figure 1. It is important to note these 486 records included those with responses of “ignored”, which is an option for all response fields in the SARS form. In addition, there was a high frequency of incomplete information on the SARS forms. Responses of “unknown” and missing responses were not included in the statistical analyses, thus altering the sample size for each variable. Even with the lack of some information, it was possible to perform data mining with robust statistical models.

In Paraíba, from April 2020 to July 2021, COVID-19 was most common in female children (n = 277; 57.0%), with a mean age of 7.3 years and a median age of 5.5 years, with a standard deviation (SD) of 7.14 years, and in patients who self-reported as mixed-race individuals (n = 326; 75.8%).

Among the signs and symptoms, the most frequent was fever (n = 298; 65.9%), and neurological problems were the most frequently reported comorbidity (n = 26; 19.1%). At admission, 55.4% (n = 246) of patients did not require ventilatory support, 58.4% (n = 171) did not undergo X-ray, 73.0% (n = 355) did not require intensive care, and 91.1% (n = 339) progressed to cure (Table 1).

**Table 1:** Demographic and clinical characteristics of children and adolescents with reported COVID-19

Variable	
Sex (n = 486)	
Female	277 (57.0)
Male	209 (43.0)
Age (n = 483) (years)	
0 - 4	209 (43.3)
5 - 9	65 (13.5)
10 - 15	76 (15.7)
16 - 18	133 (27.5)
Pregnancy (n = 478)	
Not applicable	354 (74.1)
Not pregnant	79 (16.5)
3^rd^ trimester	32 (6.7)
1^st^ trimester	8 (1.7)
2^nd^ trimester	3 (0.6)
Unknown gestational age	2 (0.4)
Race (n = 430)	
Brown	326 (75.8)
White	86 (20.0)
Black	10 (2.3)
Indigenous	5 (1.2)
Asian	3 (0.7)
Symptoms*	
Fever	298 (65.9)
Cough	241 (54.2)
Dyspnea	215 (49.7)
Respiratory distress	203 (49.0)
SatO_2_ < 95%	120 (31.7)
Sore throat	74 (20.2)
Vomiting	70 (18.7)
Diarrhea	71 (19.1)
Other respiratory symptoms	42 (33.1)
Musculoskeletal symptoms	20 (15.7)
Gastrointestinal symptoms	18 (14.2)
Neurological symptoms	41 (32.3)
Other generalized symptoms	14 (11.0)
Comorbidities*	
Neurological problems	33 (29.1)
Puerperium	24 (17.9)
Heart disease	21 (15.6)
Immunodepression	16 (12.2)
Asthma	16 (11.9)
Lung disease	9 (6.8)
Obesity	5 (3.8)
Diabetes	6 (4.6)
Hematological problems	5 (3.8)
Liver problems	6 (4.6)
Down syndrome	5 (3.7)
Kidney problems	3 (2.3)
Other syndromes	10 (14.4)
Congenital problems	8 (11.4)
Cancer	7 (10.0)
Obstetric and neonatal problems	17 (24.3)
Other conditions	22 (31.4)
ICU admission (n = 486)	
Yes	131 (27.0)
No	355 (73.0)
Ventilatory support (n = 444)	
No	246 (55.4)
Yes, noninvasive	136 (30.6)
Yes, invasive	62 (14.0)
X-ray (n = 293)	
Not performed	171 (58.4)
Normal	52 (17.7)
Interstitial infiltrate	38 (13.0)
Other image types	23 (7.8)
Mixed	7 (2.4)
Consolidation	2 (0.7)
Outcome (n = 372)	
Cure	339 (91.1)
Death	30 (8.1)
Death from other causes	3 (0.8)

*The variables symptoms and comorbidities are cumulative, i.e., a patient may have a varied combination of symptoms and comorbidities. Therefore, the percentages for these variables do not add up to 100%, as they depend on the number of symptoms and comorbidities reported by the patients.

SatO_2_ - oxygen saturation; ICU - intensive care unit.

Among the social context variables of children and adolescents, reports were predominant in the following groups: zone, urban (n = 384; 85.5%); municipality, João Pessoa (n = 139; 28.5%); municipality size, large (n = 268; 55.1%); population density, above 165.52 inhabitants/km^2^ (n = 242; 49.8%); Municipal Human Development Index (MHDI), high (n = 217; 44.7%); Social Vulnerability Index (SVI), low (n = 231; 47.5%); infant mortality rate, 10.32% - 12.92% (n = 177; 38.6%); illiteracy rate up to 18 years of age, 13.08% - 28.83% (n = 244; 50.2%); facilities with Family Health Strategy coverage, 23 - 199 (n = 236; 48.5%); number of health facilities, 34 - 208 (n = 233; 47.9%); available pediatric beds, 25 - 249 (n = 219; 45.0%); percentage of urban roads paved, 17.0% - 25.1% (n = 227; 46.6%); and sewage treatment rate, 56.2% - 70.8% (n = 164; 33.8%) (Table 2).

**Table 2:** Contextual variables of the reported patients with COVID-19

Variable	
Zone (n = 449)	
Urban	384 (85.5)
Rural	64 (14.3)
Periurban	1 (0.2)
Municipality of residence (n = 486)	
João Pessoa	139 (28.6)
Campina Grande	61 (12.6)
Mamanguape	28 (5.8)
Santa Rita	14 (2.9)
Cajazeiras	13 (2.6
Caaporã	12 (2.4)
Patos	10 (2.1)
Bayeux	9 (1.9)
Cabedelo	9 (1.9)
Monteiro	7 (1.4)
Guarabira	6 (1.2)
Cities with 5 cases	6 (6.2)
Cities with 4 cases	4 (3.2)
Cities with 3 cases	9 (5.6)
Cities with 2 cases	25 (10.3)
Cities with 1 case	55 (11.3)
Population size (n = 486)	
Large	268 (55.1)
Medium	162 (33.3)
Small	56 (11.5)
Demographic density (n = 486), inhabitants per km ^2^	
Up to 88,84	124 (25.5)
88,85 - 165,52	120 (24.7)
165,53 - 4321,28	242 (49.8)
MHDI (n = 486)	
Low	173 (35.6)
Medium	96 (19.5)
High	217 (44.7)
SVI (n = 486)	
Low vulnerability	231 (47.5)
Medium vulnerability	47 (9.7)
High vulnerability	145 (29.8)
Very high vulnerability	63 (13.0)
Infant mortality rate (n = 459), deaths per 1,000 live births	
Up to 10,31	115 (25.1)
10,32 - 12,92	177 (38.6)
12,93 - 13,55	65 (14.1)
Above 13,55	102 (22.2)
Illiteracy rate (n = 486), %	
13,08 - 28,83	244 (50.2)
28,84 - 40,34	129 (26.5)
Above 40,34	113 (23.3)
FHS coverage (n = 486), units	
Up to 8	137 (28.2)
9 - 22	113 (23.3)
23 - 199	236 (48.5)
Health care facilities (n = 486)	
Up to 12	141 (29.0)
13 - 33	112 (23.1)
34 - 208	233 (47.9)
Total pediatric beds (n = 486)	
Up to 4	134 (27.5)
5 - 24	133 (27.4)
25 - 249	219 (45.1)
Paved urban roads (n = 484), %	
Up to 3,9	136 (28.1)
4,0 - 16,9	109 (22.5)
17,0 - 25,1	227 (46.9)
Above 25,1	12 (2.5)
Sewage treatment (n = 485), %	
Up to 21,1	127 (26.2)
21,2 - 56,1	118 (24.3)
56,2 - 70,8	164 (24.3)
Above 70,8	76 (15.7)
Gini index (n = 486), per capita household income	
Up to 0,5182	122 (25.1)
0,5183 - 0,5640	132 (27.2)
0,5641 - 0,6290	223 (45.9)
Above 0,6291	9 (1.8)

FHS - Family Health Strategy. MHDI - Municipal Human Development Index; SVI - Social Vulnerability Index.

In the statistical models developed, some variables showed better fit in the logistic regression model as continuous variables, including age and the Gini index. In the individual tests of explanatory variables with the outcome, the Gini index as a categorical variable (categories defined by quartiles) had a p value of 0.332 and was not considered for the next analysis. Age as a categorical variable (categories defined by quartiles) had a p value < 0.20 in the single-variable test; however, in the adjusted regression model, it was not statistically significant (p values: 0 to 4 years old = 1; 4 - 9 years old = 0.187; 9 - 15 years old = 0.3343; 15 - 18 years old = 0.111), considering the final model α of 5%. The population size showed the best fit in the logistic regression as a categorical variable, and in the multilevel logistic regression, it remained in the final model as a continuous variable.

In the bivariate analyses of the simple logistic regressions, the variables with p values ≤ 0.20 were included in the multiple logistic regression model (Table 3). The results of the final multiple logistic regression model are shown in table 4. With increasing age, the odds of ICU admission decreased by 6% (odds ratio - OR = 0.935; confidence interval - 95%CI 0.901 - 0.971). Regarding sex, male children and adolescents had a 98% greater chance of receiving intensive care (OR = 1.981; 95%CI 1.181 - 3.322) than female children and adolescents. Cough and fever were symptoms that reduced the likelihood of hospitalization in the ICU by 68% (OR = 0.322; 95%CI 0.175 - 0.593) and 58% (OR = 0.415; 95%CI 0.234 - 0.737), respectively. Patients with respiratory distress and dyspnea were 2.43 (OR = 2.428; 95%CI 1.293 - 4.562) and 3.56 (OR = 3.565; 95%CI 1.771 - 7.175) times more likely, respectively, to require intensive care than patients who did not report these symptoms.

**Table 3:** Bivariate analysis of hospitalization of children and adolescents with COVID-19 in the intensive care unit

Variable	p value	OR	95%CI
Sex			
Female	-	1	-
Male	0.009	1.709	1.142 - 2.563
Age (years)	0.001	0.999	0.9997 - 0.9999
0 - 4	-	1	-
5 - 9	0.022	0.456	0.225 - 0.869
10 - 15	0.007	0.411	0.209 - 0.767
16 - 18	0.008	0.509	0.305 - 0.832
Regional health of the residential area			
I NRS João Pessoa	-	1	-
III NRS Campina Grande	0.134	1.495	0.877 - 2.519
IV NRS Cuite	0.136	6.290	0.593 - 136.718
V NRS Monteiro	0.078	3.145	0.849 - 11.654
XI NRS Princess Isabel	0.093	4.718	0.765 - 36.430
Population size			
Small	-	1	-
Large	0.201	1.589	0.805 - 3.382
Gini index	0.118	0.065	0.002 - 1.989
Cough			
No	-	1	-
Yes	0.014	0.592	0.389 - 0.897
Fever			
No	-	1	-
Yes	0.006	0.548	0.357 - 0.841
Sore throat			
No	-	1	-
Yes	0.069	0.564	0.295 - 1.024
Dyspnea			
No	-	1	-
Yes	2.52e-05	2.533	1.647 - 3.939
Respiratory distress			
No	-	1	-
Yes	4.8e-07	3.148	2.022 - 4.973
Diarrhea			
No	-	1	-
Yes	0.071	0.558	0.286 - 1.026
SatO_2_ < 95%			
No	-	1	-
Yes	1.14e-06	3.156	1.986 - 5.041
Vomiting			
No	-	1	-
Yes	0.027	0.472	0.232 - 0.472
Other symptoms			
No	-	1	-
Yes	0.0270	0.559	0.331 - 0.928
Presence of any risk factor			
No	-	1	-
vYes	0.016	0.599	0.367 - 0.711
Puerperal woman			
No	-	1	-
Yes	0.017	0.214	0.049 - 0.669
Asthma			
No	-	1	-
Yes	0.078	0.252	0.038 - 0.960
Neurological problems			
No	-	1	-
Yes	0.056	2.333	0.973 - 5.622
X-ray			
Normal	-	1	-
Interstitial infiltrate	0.061	2.431	0.967 - 6.2960
Other images	0.022	3.417	1.197 - 10.027

OR - odds ratio; 95%CI - 95% confidence interval; NRS - Regional Health Center.

**Table 4:** Final logistic regression model adjusted for factors associated with hospitalization of children and adolescents with COVID-19 in the intensive care unit

Variable	p value	OR	95%CI
Intercept	0.189	5.104	0.446 - 58.345
Age	0.000	0.935	0.901 - 0.971
Sex			
Female	-	1	-
Male	0.009	1.981	1.181 - 3.322
Cough			
No	-	1	-
Yes	0.000	0.322	0.175 - 0.593
Fever			
No	-	1	-
Yes	0.003	0.415	0.234 - 0.737
Respiratory distress			
No	-	1	-
Yes	0.006	2.428	1.293 - 4.562
Dyspnea			
No	-	1	-
Yes	0.000	3.565	1.771 - 7.175
Population size			
Small	-	1	-
Large	0.035	2.696	1.074 - 6.767
Gini index	0.010	0.003	0.000 - 0.243

OR - odds ratio; 95%CI - 95% confidence interval.

Regarding population size, children and adolescents with COVID-19 residing in large cities were 2.70 (OR = 2.696; 95%CI 1.074 - 6.767) times more likely to be admitted to the ICU than were patients from small or medium cities. The Gini index exerted a substantial influence on the outcome; as the value of this coefficient increased, there was a marked decrease of 99% (OR = 0.003; 95%CI 0.000 - 0.243) in the chance of hospitalization in the ICU of children and adolescents with COVID-19 (Table 4).

The ROC curve indicated a good fit of the model, with an area under the curve of 0.799 or 79%, a value that was considered the cutoff point. The sensitivity (true positives) was 81.4%, and the specificity (false positives) was 67.5%.

For the multilevel regression model, the ICC was 0.146, indicating that 15% of the chance of hospitalization in the ICU for children and adolescents with COVID-19 in Paraíba was explained by the characteristics of the municipality. Next, the independent variables were tested with the response variable, and the final model was obtained, considering an α of 0.05; the results are shown in table 5.

**Table 5:** Final multilevel logistic regression model adjusted to evaluate the association of individual and contextual factors with the outcome of hospitalization in the intensive care unit of children and adolescents with COVID-19

Variable	p value	OR	95%CI
Intercept	< 2 and - 16	5.104	0.446 - 58.345
Sex			
Female	-	1	-
Male	< 2e - 16	1.694	1.680 - 1.707
Race			
Nonbrown	-	1	-
Brown	2.36e - 16	0.981	0.973 - 0.989
Gini index	0.0493	0.022	0.021 - 0.022
Population size	3.93e - 15	1.019	1.011 - 1.027
Dyspnea			
No	-	1	-
Yes	0.071	0.996	0.988 - 1.004

OR - odds ratio; 95%CI - 95% confidence interval.

In this multilevel analysis, male children and adolescents were 1.69 times more likely to be admitted to the ICU (OR = 1.69; 95%CI 1.68 - 1.71) than female patients were. Patients who self-reported as mixed race had a 2% lower chance (OR = 0.98; 95%CI 0.97 - 0.99) of needing intensive care than those who did not. This variable was not included in the non-multilevel regression model (p value > 0.20) because it presented p values of 0.96, 0.59, 0.98, 0.98 and 1.00 for patients of brown, black, Asian, indigenous and white races, respectively. In addition, the following frequencies of patients with respect to race (n = 430) admitted to the ICU were observed: mixed-race, 28.2% (n = 92/326); non-brown, 25% (26/104). As the Gini index increased, the chance of ICU admission decreased by 98% (OR = 0.02; 95%CI 0.02 - 0.02). The growth in population size per 100,000 inhabitants increased the likelihood of referring children and adolescents with COVID-19 to critical care by 1.02 times (OR = 1.02; 95%CI 1.01 - 1.03). The dyspnea variable was important for model fit, but it was not significant at the 5% level.

The *deviance* value obtained for the model was 479.432, indicating a good fit. In addition to the deviance, the ROC curve was generated, with an area under the curve of 0.691 (95%CI 0.637 - 0.743), indicating good quality of the model.

## DISCUSSION

The statistical models developed show that the hospitalization of children and adolescents with COVID-19 in the ICU in the state of Paraíba was associated with variables specific to the individual and to the social context of the patients. These findings highlight the social nature of the disease and reinforce the need to consider contextual determinants that may influence the health status of children and adolescents among the variables of interest to be analyzed. In addition, the multilevel model showed differences in the estimates of the parameters compared to other types of statistical models, indicating that this type of modeling is relevant. The multiple logistic regression model without hierarchical levels indicated that age, male sex, cough, fever, respiratory distress, dyspnea, population size and the Gini index were variables that influence the hospitalization of children and adolescents in the ICU. Other studies have used logistic regression to assess the relationships between contextual variables and outcomes.^(11.12)^ In the multilevel regression, sex, race, the Gini index and population size were entered into the final model and were found to influence the outcome.

The analysis revealed that as age increased, there was a reduction in the chance of these patients requiring intensive care. Younger individuals seem to be affected more severe clinical manifestations of COVID-19. Younger children had a greater frequency of hospitalization and need for the ICU than older children did.^(13)^ Greater sensitivity to dehydration and incomplete vaccination are potential factors that may increase the risk of complications from COVID-19 in children under 1 year of age, according to an Iranian study.^(14)^

According to both statistical models, male children and adolescents were more likely to need intensive care. Some studies have shown differences in the occurrence of COVID-19 between sexes.^(15-17)^ A multicenter study conducted in 19 ICUs in Brazil showed that the majority of hospitalized patients were male,^(18)^ which is consistent with these results.

Studies of adult patients have shown that there are biological differences between men and women, such as the expression of angiotensin-converting enzyme (ACE2) and transmembrane serine protease 2 (TMPRSS2), which are responsible for the entry of severe acute respiratory syndrome coronavirus 2 (SARS-CoV-2) into cells and viral invasion, and the regulation of these proteins by sex hormones may be responsible for the greater lethality of COVID-19 in the male population.^(15.16)^ It is believed that these hypotheses also explain the higher frequency of more severe conditions in boys, but there are few studies focused on children that reveal this predominance.

According to the logistic regression results, pediatric patients who presented with cough and fever were less likely to be admitted to the ICU. These symptoms are defined as some of the warning signs in patients with suspected COVID-19 according to the *Agência Nacional de Vigilância Sanitária* (ANVISA ),^(19)^ and the appearance of these symptoms at the onset of infection may lead individuals to seek early medical care, with chances of minimizing the worsening of the condition.

Symptoms of respiratory distress and dyspnea were considered factors that contributed to the occurrence of the outcome. These findings indicated that children and adolescents with symptoms of respiratory tract infection were more likely to require ICU admission.^(20)^ Shortness of breath has been significantly associated with the severity of COVID-19.^(21)^ Dyspnea has been reported as the most common finding in severe COVID-19 cases in neonates.^(22)^ Vitamin D deficiency has been reported as one of the causes of dyspnea in hospitalized Iranian children with severe COVID-19.^(14)^

Patients who self-reported as mixed race were less likely to be admitted to the ICU than those who did not. These findings are contradictory to those reported in the literature.^(23-25)^ Moreover, the majority of ICU admissions in the present study were mixed-race patients. Throughout the pandemic period, new hypotheses emerged to explain the relationship between race and COVID-19, such as the identification of blood types with greater chances of infection by SARS-CoV-2 that are more common in white and Hispanic individuals.^(26)^

This sample included more mixed-race individuals compared to other races, which may explain why mixed race/color was a protective factor for the hospitalization of children and adolescents in the ICU. Patients who self-reported as mixed race were also more likely to progress to a cure in this study. This finding may be a reflection of the fact that the Continuous Quarterly National Household Sample Survey (Continuous PNAD - *Pesquisa Nacional por Amostra de Domicílios Contínua Trimestral*)^(27)^ identified that the population of Paraíba is predominantly mixed-race (59.6%). Another explanation for the results may lie in the stratification of the database, which separated the individuals into mixed-race, white, black, Asian and indigenous individuals and did not group black and mixed-race individuals into the same group. This analysis considered only two groups: patients who did and did not self-report as mixed race. In addition, the data mining of the hierarchical level analysis showed the influence of the *clusters* (municipalities) on the outcome, which was not observed in the single-level regression model. Therefore, the multilevel model included some explanatory variables that were different from the single-level regression model, including race. Therefore, despite the statistical significance of mixed race/color, this result needs to be analyzed with caution, as studies^(23-25)^ have reported the unfavorable repercussions of COVID-19 in mixed-race individuals.

Regarding population size, in the single-level and multilevel logistic regression models, children and adolescents with COVID-19 residing in large cities were more likely to be admitted to the ICU. Ceará and Piauí also reported higher rates of disease spread in their capitals and neighboring municipalities, which was explained by the high population density contributing to the rapid spread of the virus.^(28.29)^ The spread of infectious diseases caused by viruses is closely linked to the displacement of people, urbanization and the movement of foreigners, which are characteristics inherent to large metropolises.^(30)^ In addition, municipalities with more than 400 thousand inhabitants have higher levels of *per capita* health expenditures and higher values of the transfer of the *Sistema Único de Saúde* (SUS) and direct more of their own revenues to health. In addition, as the population grows, these municipalities assume significant roles as a regional reference to serve the community, incorporating procedures of medium and high complexity,^(31)^ consequently increasing the number of hospitalizations in intensive care beds.

The Gini index appeared in both models and exhibited an inverse relationship, decreasing the chances of ICU admission as its values increased. An increased Gini index value indicates greater inequality in income distribution. A high Gini index may result in the distancing of population segments with little chance of social integration, initially interrupting the increase in SARS-CoV-2 transmission and decreasing the spread of the virus.^(32)^

The use of two statistical methods with and without hierarchical levels for data modeling reinforced the relationship of the predictor variables obtained in the final models influencing the same outcome. Logistic regression without hierarchical levels highlighted the relationships of explanatory variables with the hospitalization of children and adolescents with COVID-19 in the ICU in a more general way, without considering the characteristics that may exist in different municipalities. The multilevel model, on the other hand, allows for the exploration of the data in more detail, indicating the variability in the outcome between levels. Therefore, the incorporation of the random effects of the groups, municipalities in this study, was relevant for the estimation of the parameters when the responses were grouped.

Both statistical models provided important information about the event of interest, and analyzing the contribution of results presented by different models may improve the understanding of how children and adolescents with COVID-19 progress to ICU admission, considering aspects that are more comprehensive and common to certain groupings, favoring the development of more assertive strategies.

These results should be interpreted with caution, as these are secondary data with a risk of underreporting. In addition, difficulties in accessing health services and testing the population, especially children and adolescents, may underestimate the real number of cases of the disease, which is a limitation of this study. Another limitation is related to the variables collected in public databases, such as the Department of Informatics of the SUS (DATASUS) and the Atlas of Social Vulnerability, which have not been recently updated, impairing comparisons with current information, as many children and adolescents were born years after the publication of these data. Despite these limitations, the results of this study suggest the need to consider contextual variables to better understand the course of COVID-19 in children and adolescents who require intensive care.

## CONCLUSION

The association between patient characteristics and a severity of SARS-CoV-2 infection resulting in the need for intensive care may be influenced by the social and economic context in which children and adolescents live, as well as the magnitude of these factors. These effects are estimated more accurately with the inclusion of a multilevel regression model in the analyses.

Thus, we suggest that the clinical and socioeconomic profiles of the population may guide the development of policies to combat the coronavirus, making it necessary to carefully look at children and adolescents, among whom the number of cases and deaths has been increasing over time. This pandemic is a very unstable scenario, with the emergence of new variants and the restriction of vaccines that meet the needs of all children, especially the youngest age group, which is most strongly affected. Conducting strategies through the prism of clinical and social realities is likely more useful for controlling and mitigating SARS-CoV-2 in this population.

## References

[1] Shen Q, Li J, Zhang Z, Guo S, Wang Q, An X (2022). COVID-19: systemic pathology and its implications for therapy. Int J Biol Sci.

[2] Quantin C, Tubert-Bitter P (2022). COVID-19 and social inequalities: a complex and dynamic interaction. Lancet Public Health.

[3] Nikolopoulou GB, Maltezou HC (2022). COVID-19 in children: where do we stand?. Arch Med Res.

[4] Martins-Filho PR, Quintans-Júnior LJ, de Souza Araújo AA, Sposato KB, Souza Tavares CS, Gurgel RQ (2021). Socio-economic inequalities and covid-19 incidence and mortality in Brazilian children: a nationwide register-based study. Public Health.

[5] Instituto Brasileiro de Geografia e Estatística (IBGE) (2021). IBGE Cidades e Estados do Brasil.

[6] Brasil, Ministério da Saúde, DATASUS (2021). Brasil.

[7] Instituto de Pesquisa Econômica Aplicada (IPEA) (2018). A nova plataforma de vulnerabilidade social: primeiros resultados do índice de vulnerabilidade social para a série histórica da PNAD (2011-2015) e desagregações por sexo, cor, e situação de domicílios.

[8] Brasil, Programa das Nações Unidas para o Desenvolvimento (PNUD Brasil) (2020). Atlas de desenvolvimento humano no Brasil.

[9] Fávero LP, Belfiore PP, Silva FL, Chan BL (2009). Análise de dados: modelagem multivariada para tomada de decisões.

[10] R Core Team (2021). R: A language and environment for statistical computing.

[11] Braga LH, Menezes CS, Martins IV, Silva JD, Torres JL (2022). Fatores associados à piora no estilo de vida durante a pandemia de COVID-19 na população brasileira de lésbicas, gays, bissexuais, transexuais, travestis e identidades relacionadas: estudo transversal. Epidemiol Serv Saúde.

[12] Franck MC, Monteiro MG, Limberger RP (2020). Mortalidade por suicídio no Rio Grande do Sul: uma análise transversal dos casos de 2017 e 2018. Epidemiol Serv Saúde.

[13] Cavalcante AN, Tavares LV, Bastos ML, Almeida RL (2021). Clinical-epidemiological profile of children and adolescents with COVID-19 in Ceará. Rev Bras Saude Mater Infant.

[14] Madani S, Shahin S, Yoosefi M, Ahmadi N, Ghasemi E, Koolaji S (2021). Red flags of poor prognosis in pediatric cases of COVID-19: the first 6610 hospitalized children in Iran. BMC Pediatr.

[15] Haitao T, Vermunt JV, Abeykoon J, Ghamrawi R, Gunaratne M, Jayachandran M (2020). COVID-19 and sex differences: mechanisms and biomarkers. Mayo Clin Proc.

[16] Márquez EJ, Trowbridge J, Kuchel GA, Banchereau J, Ucar D (2020). The lethal sex gap: COVID-19. Immun Ageing.

[17] Spaulding AB, Watson D, Norton L (2022). Inpatient and outpatient differences in pediatric patients with laboratory-confirmed COVID-19. Pediatr Infect Dis J.

[18] Prata-Barbosa A, Lima-Setta F, Santos GR, Lanziotti VS, de Castro RE, de Souza DC, Raymundo CE, de Oliveira FR, de Lima LF, Tonial CT, Colleti J, Bellinat AP, Lorenzo VB, Zeitel RS, Pulcheri L, Costa FC, La Torre FP, Figueiredo EA, Silva TP, Riveiro PM, Mota IC, Brandão IB, de Azevedo ZM, Gregory SC, Boedo FRO, de Carvalho RN, Castro NA, Genu DH, Foronda FK, Cunha AJL, de Magalhães-Barbosa MC, Brazilian Research Network in Pediatric Intensive Care, (BRnet-PIC) (2020). Pediatric patients with COVID-19 admitted to intensive care units in Brazil: a prospective multicenter study. J Pediatr (Rio J).

[19] Brasil, Ministério da Saúde, Agência Nacional de Vigilância Sanitária (ANVISA) (2021). Nota Técnica GVIMS/GGTES/ANVISA Nº 04/2020. Orientações para Serviços de Saúde: Medidas de Prevenção e Controle que devem ser adotadas durante a assistência aos casos suspeitos ou confirmados de Infecção pelo Novo Coronavírus (Sars-Cov-2) - atualizada em 25/02/2021.

[20] Götzinger F, Santiago-García B, Noguera-Julián A, Lanaspa M, Lancella L, Calò Carducci FI, Gabrovska N, Velizarova S, Prunk P, Osterman V, Krivec U, Lo Vecchio A, Shingadia D, Soriano-Arandes A, Melendo S, Lanari M, Pierantoni L, Wagner N, L'Huillier AG, Heininger U, Ritz N, Bandi S, Krajcar N, Roglic S, Santos M, Christiaens C, Creuven M, Buonsenso D, Welch SB, Bogyi M, Brinkmann F, Tebruegge M, ptbnet COVID-19 Study Group (2020). COVID-19 in children and adolescents in Europe: a multinational, multicentre cohort study. Lancet Child Adolesc Health.

[21] Sedighi I, Fahimzad A, Pak N, Khalili M, Shokrollahi MR, Heydari H (2022). A multicenter retrospective study of clinical features, laboratory characteristics, and outcomes of 166 hospitalized children with coronavirus disease 2019 (COVID-19): a preliminary report from Iranian Network for Research in Viral Diseases (INRVD). Pediatr Pulmonol.

[22] Liguoro I, Pilotto C, Bonanni M, Ferrari ME, Pusiol A, Nocerino A (2020). SARS-COV-2 infection in children and newborns: a systematic review. Eur J Pediatr.

[23] Araújo EM, Caldwell KL, Santos MP, Souza IM, Santa Rosa PL, Santos AB (2020). Morbimortalidade pela Covid-19 segundo raça/cor/etnia: a experiência do Brasil e dos Estados Unidos. Saúde Debate.

[24] Aragão HT, Santana JT, Silva GM, Santana MF, Silva LN, Oliveira ML (2022). Impactos da Covid-19 à luz dos marcadores sociais de diferença: raça, gênero e classe social. Saúde Debate.

[25] Baqui P, Bica I, Marra V, Ercole A, van der Schaar M (2020). Ethnic and regional variations in hospital mortality from COVID-19 in Brazil: a cross-sectional observational study. The Lancet Glob Health.

[26] Luo J, Craver A, Zakin P, Stepniak L, Moore K, King J (2022). Race may modify the association between blood type and COVID-19 infection. EJHaem.

[27] Instituto Brasileiro de Geografia Estatística (IBGE) Sistema IBGE de Recuperação Automática - SIDRA - Banco de tabelas de estatísticas. Pesquisa Nacional por Amostra de Domicílios Contínua Trimestral. Tabela 6403: População, por cor ou raça.

[28] Maciel JA, Castro-Silva II, Farias MR (2020). Análise inicial da correlação espacial entre a incidência de COVID-19 e o desenvolvimento humano nos municípios do estado do Ceará no Brasil. Rev Bras Epidemiol.

[29] Silva VR, Pacheco ES, Cardoso OO, Lima LH, Rodrigues MT, Mascarenhas MD (2022). Tendência temporal das taxas de incidência e de mortalidade por COVID-19 e sua relação com indicadores socioeconômicos no Piauí: estudo ecológico, 2020-2021. Epidemiol Serv Saúde.

[30] Jo Y, Hong A, Sung H (2021). Density or connectivity: what are the main causes of the spatial proliferation of COVID-19 in Korea?. Int J Environ Res Public Health.

[31] Lui L, Lima LL, Aguiar R, Machado JA, Albert C (2022). A potência do SUS no enfrentamento à Covid-19: alocação de recursos e ações nos municípios brasileiros. Trab Educ Saúde.

[32] Kong JD, Tekwa EW, Gignoux-Wolfsohn SA (2021). Social, economic, and environmental factors influencing the basic reproduction number of COVID-19 across countries. PloS One.

