# Supplementary material for: Analysis of factors associated with admission to the intensive care unit of children and adolescents with COVID-19: application of a multilevel model
Source: Crit Care Sci. 2024 Jul 1;36:e20240068en. doi: 10.62675/2965-2774.20240068-en (PMC11239204; doi:10.62675/2965-2774.20240068-en)
Supplement: Translation

# Translation: Análise dos fatores associados ao internamento na unidade de terapia intensiva de crianças e adolescentes com COVID-19: aplicação de um modelo multinível

**DOI:** 10.62675/2965-2774.20240068-pt

**Authors:** Lecidamia Cristina Leite Damascena, Aline Roseane Queiroz de Paiva Faria, Nyellisonn Nando Nóbrega de Lucena, Ana Hermínia Andrade e Silva, Talita Tavares Alves de Almeida, Diana de Fátima Alves Pinto, Hemílio Fernandes Campos Coêlho, Ana Maria Gondim Valença

**Affiliations:** 1 Brasil Programa de Pós-Graduação em Modelos de Decisão e Saúde, Centro de Ciências Exatas e da Natureza, Universidade Federal da Paraíba - João Pessoa (PB), Brasil.; 2 Brasil Secretaria de Saúde do Estado da Paraíba - João Pessoa (PB), Brasil.

**Keywords:** COVID-19, Infecções por coronavírus, Criança, Adolescente, Modelos logísticos, Análise multinível, Vulnerabilidade social, Unidades de terapia intensiva pediátrica

## Abstract

**Objetivo:**

Identificar fatores associados ao internamento na unidade de terapia intensiva de crianças e adolescentes com COVID-19.

**Método:**

Estudo de coorte retrospectiva, com dados secundários, de crianças e adolescentes hospitalizados (zero a 18 anos), notificados com COVID-19 na Paraíba, de abril de 2020 a julho de 2021, totalizando 486 registros. Foram realizadas análise descritiva, regressão logística e regressão multinível, considerando o nível de significância de 5%.

**Resultados:**

Na regressão logística sem níveis hierárquicos, ocorreu aumento da chance de internamento na unidade de terapia intensiva em pacientes do sexo masculino (RC = 1,98; IC95% 1,18 - 3,32), com desconforto respiratório (RC = 2,43; IC95% 1,29 - 4,56), dispneia (RC = 3,57; IC95% 1,77 - 7,18) e residentes em cidades com grande porte populacional (RC = 2,70; IC95% 1,07 - 6,77). Foi observada diminuição da chance de cuidados intensivos com aumento da idade em anos (RC = 0,94; IC95%=0,90 - 0,97), presença de tosse (RC = 0,32; IC95% 0,18 - 0,59), febre (RC = 0,42; IC95% 0,23 - 0,74) e aumento no Índice de Gini (RC = 0,003; IC95% 0,000 - 0,243). Na análise multinível, a chance de internamento na unidade de terapia intensiva aumentou no sexo masculino (RC = 1,70; IC95%=1,68-1,71) e por conta do aumento no porte populacional do município a cada 100 mil habitantes (RC = 1,01; IC95% 1,01 - 1,03); a chance de internamento na unidade de terapia intensiva diminuiu em pacientes pardos *versus* não pardos (RC = 0,981; IC95% 0,97 - 0,99) e por conta do aumento a cada pontuação do Índice de Gini (RC = 0,02; IC95% 0,02 - 0,02).

**Conclusão:**

Os efeitos das condições próprias do paciente e do contexto social na necessidade de cuidados intensivos em crianças e adolescentes com infecção pelo SARS-CoV-2 são mais bem estimados com a inclusão de um modelo de regressão multinível nas análises.

## INTRODUÇÃO

A doença causada pelo coronavírus 2019 (COVID-19) provocou sequelas inimagináveis na saúde pública e a perda de milhares de vidas.^(1)^ Diversos países se mobilizaram para encontrar estratégias de controle e combate à COVID-19, que se tornou uma emergência de saúde pública.

Nos países de baixa e média renda, a incidência da COVID-19 pode ser influenciada pela vulnerabilidade social apresentada por algumas classes menos favorecidas. As populações vulneráveis dispõem de características e comportamentos específicos relacionados à maior exposição ao vírus, sendo mais suscetíveis à infecção, à maior frequência de associação entre comorbidades e desfechos desfavoráveis e à desigualdade no acesso aos cuidados em saúde.^(2)^

As crianças e os adolescentes apresentam quadros menos severos da COVID-19.^(3)^ Contudo, também foram afetadas e tiveram consequências diretas do isolamento. A pandemia foi associada a profundas alterações educacionais, sociais e psicológicas e à insegurança alimentar, aumentando o risco de resultados adversos graves que podem provocar mais mortes de crianças e adolescentes nas regiões mais carentes.^(4)^

Nesse sentido, torna-se relevante investigar se existem determinantes de vulnerabilidade social, em nível individual e contextual, que conduzem a desfechos desfavoráveis de crianças e adolescentes infectados pela COVID-19. O objetivo deste trabalho foi identificar fatores associados ao internamento na unidade de terapia intensiva (UTI) de crianças e adolescentes com COVID-19.

## MÉTODOS

Trata-se de um estudo de coorte retrospectiva, exploratória, com abordagem quantitativa, no intuito de identificar se existem fatores associados ao internamento na UTI de crianças e adolescentes da Paraíba com COVID-19. A Paraíba é um dos estados da Região Nordeste do Brasil e apresenta população estimada de 4.039.277 habitantes, densidade demográfica de 66,70 hab/km^2^, Índice de Desenvolvimento Humano (IDH) de 0,658 e Índice de Gini 0,559. Possui taxa de mortalidade infantil de 13,29 óbitos por mil nascidos vivos.^(5)^

A população do estudo foi composta de crianças e adolescentes de zero a 18 anos, notificados por meio da ficha de síndrome respiratória aguda grave (SRAG), que foram hospitalizados e tiveram diagnóstico final da COVID-19, no período de abril de 2020 a julho de 2021. O banco de dados foi disponibilizado pela Secretaria de Saúde do Estado da Paraíba (SES-PB), com informações de abril de 2020 a julho de 2021.

As variáveis individuais referentes aos dados sociodemográficos, sinais e sintomas foram extraídos da planilha disponibilizada pela SES-PB, e as variáveis contextuais foram obtidas por meio de sítios eletrônicos.^(5-8)^ A categorização foi realizada para algumas variáveis, como porte populacional, que se distribuiu em pequeno (menos de 10.000 habitantes), médio (entre 10.000 e 50.000 habitantes) e grande (acima de 50.000 habitantes);^(5)^ Índice de Vulnerabilidade Social;^(7)^ e Índice de Desenvolvimento Humano Municipal.^(8)^ Outras variáveis numéricas foram categorizadas utilizando a distribuição interquartil; são elas: idade, densidade demográfica, Índice de Gini, cobertura da Estratégia Saúde da Família, total de leitos pediátricos, estabelecimentos de saúde, mortalidade infantil, esgotamento sanitário e pavimentação de vias urbanas.

A fim de esgotar as possibilidades de utilização das variáveis, todas foram testadas na forma categórica e numérica nas duas modelagens estatísticas, contudo, algumas não permaneceram nos modelos ajustados.

Inicialmente, realizou-se a análise descritiva e, na modelagem dos dados, aplicou-se o modelo de regressão logística, realizando, a princípio, o cruzamento de cada covariável com o evento de interesse. Em seguida, foram incluídas no modelo as associações entre as variáveis explicativas e o desfecho com valores de p ≤ 0,20. Foi considerada a utilização do valor de p de 0,20 nos testes iniciais da regressão logística. Foi aplicada a técnica *stepwise* para obtenção do modelo de regressão logística final ajustado, levando em consideração o nível de significância de 5%.^(9)^ Utilizou-se a *receiver operating characteristic curve* (curva ROC) para avaliar a qualidade do modelo final ajustado.

Utilizou-se, em seguida, um modelo de regressão logística multinível. Para esse modelo, também foi aplicado o nível de significância de 5%. Na modelagem multinível, foram considerados dois níveis hierárquicos: os indivíduos notificados com COVID-19 (nível 1) e os municípios que realizaram as notificações (nível 2). A análise multinível visa separar os efeitos dentro de cada município (à medida que algumas características das crianças e adolescentes estão relacionadas à chance de internamento na UTI) dos efeitos entre os *clusters* (características dos municípios que podem estar associadas ao desfecho), considerando o mesmo desfecho da regressão logística sem níveis hierárquicos.

As etapas iniciais para a determinação do modelo de regressão multinível foram seguidas com a centralização das variáveis preditoras e a execução de um modelo vazio, isto é, sem variáveis explicativas, para saber se a chance de internamento na UTI de crianças e adolescentes com COVID-19 era diferente entre os municípios de notificação. Calculou-se o coeficiente de correlação intraclasse (CCI), que quantifica a homogeneidade do resultado entre os *clusters*, representando a proporção de variação entre os municípios. O CCI foi obtido pela razão entre a variância dos resíduos do nível municipal e a soma entre as variâncias do nível municipal e individual. Após esse procedimento, testaram-se os modelos com as variáveis explicativas. A princípio, as associações consideradas significantes para a regressão logística entraram no modelo multinível, contudo, outras variáveis foram testadas para obtenção do melhor modelo, considerando o nível de significância de 5%. Avaliaram-se o valor da *deviance* para a escolha do melhor modelo e a curva ROC para analisar a qualidade do ajuste do modelo. Para a obtenção das análises estatísticas, utilizou-se o *software* gratuito, livre e de domínio público R, versão 4.1.1.^(10)^

Este trabalho recebeu a anuência da SES-PB e foi aprovado pelo Comitê de Ética do Centro de Ciências da Saúde da Universidade Federal da Paraíba com respectivo CAAE: 39914320.2.0000.5188.

## RESULTADOS

Das 1.955 notificações de SRAG, 552 tiveram diagnóstico final confirmado da COVID-19. Destes, foram excluídos 66 registros com muitos dados faltantes, resultando em amostra de 486 casos, conforme pode ser visualizado na figura 1. Importante destacar que foram considerados 486 registros, incluindo aqueles que apresentaram também nas suas respostas a opção “ignorado”, uma vez que, na ficha de SRAG, em todos os campos de resposta, existia essa alternativa. Além disso, houve frequência elevada de preenchimento incompleto de algumas informações nas fichas de SRAG. A categoria “ignorado” e a ausência de resposta não foram incluídas nas análises estatísticas, alterando o número amostral para cada variável. Mesmo com falta de algumas informações, foi possível realizar a mineração dos dados com modelos estatísticos robustos.

**Figura 1:** Fluxo da triagem dos dados.

Na Paraíba, no período de abril de 2020 a julho de 2021, predominaram os registros da COVID-19 em crianças e adolescentes do sexo feminino (n = 277; 57,0%), com idade média de 7, 3 anos, mediana de 5,5 anos, com desvio-padrão (DP) de 7,14 anos e que se autodeclararam pardos (n = 326; 75,8%).

Entre os sinais e sintomas, o mais frequente foi a febre (n = 298; 65,9%); e os problemas neurológicos foram as comorbidades mais referidas (n = 26; 19,1%). Na internação, 55,4% (n = 246) não necessitaram de suporte ventilatório, 58,4% (n = 171) não realizaram raio X, 73,0% (n = 355) não precisaram de cuidados intensivos e 91,1% (n = 339) evoluíram para a cura (Tabela 1).

**Tabela 1:** Características demográficas e clínicas de crianças e adolescentes notificados com COVID-19

**Variável**	
Sexo (n = 486)	
Feminino	277 (57,0)
Masculino	209 (43,0)
Idade (n = 483) (anos)	
0 - 4	209 (43,3)
5 - 9	65 (13,5)
10 - 15	76 (15,7)
16 - 18	133 (27,5)
Gestante (n = 478)	
Não se aplica	354 (74,1)
Não está gestante	79 (16,5)
3º trimestre	32 (6,7)
1º trimestre	8 (1,7)
2º trimestre	3 (0,6)
Idade gestacional ignorada	2 (0,4)
Raça (n = 430)	
Pardo	326 (75,8)
Branco	86 (20,0)
Preto	10 (2,3)
Indígena	5 (1,2)
Amarelo	3 (0,7)
Sintomas*	
Febre	298 (65,9)
Tosse	241 (54,2)
Dispneia	215 (49,7)
Desconforto respiratório	203 (49,0)
SatO_2_ < 95%	120 (31,7)
Dor de garganta	74 (20,2)
Vômito	70 (18,7)
Diarreia	71 (19,1)
Outros sintomas respiratórios	42 (33,1)
Sintomas musculoesqueléticos	20 (15,7)
Sintomas gastrintestinais	18 (14,2)
Sintomas neurológicos	41 (32,3)
Outros sintomas generalizados	14 (11,0)
Comorbidades*	
Problemas neurológicos	33 (29,1)
Puerpério	24 (17,9)
Cardiopatia	21 (15,6)
Imunodepressão	16 (12,2)
Asma	16 (11,9)
Pneumopatia	9 (6,8)
Obesidade	5 (3,8)
Diabetes	6 (4,6)
Problemas hematológicos	5 (3,8)
Problemas hepáticos	6 (4,6)
Síndrome de Down	5 (3,7)
Problemas renais	3 (2,3)
Outras síndromes	10 (14,4)
Problemas congênitos	8 (11,4)
Câncer	7 (10,0)
Problemas obstétricos e neonatais	17 (24,3)
Outras condições	22 (31,4)
Internamento na UTI (n = 486)	
Sim	131 (27,0)
Não	355 (73,0)
Suporte ventilatório (n = 444)	
Não	246 (55,4)
Sim, não invasivo	136 (30,6)
Sim, invasivo	62 (14,0)
Raio X (n = 293)	
Não realizado	171 (58,4)
Normal	52 (17,7)
Infiltrado intersticial	38 (13,0)
Outros tipos de imagem	23 (7,8)
Misto	7 (2,4)
Consolidação	2 (0,7)
Evolução (n = 372)	
Cura	339 (91,1)
Óbito	30 (8,1)
Óbito por outras causas	3 (0,8)

*As variáveis sintomas e comorbidades são cumulativas, ou seja, um paciente pode apresentar uma combinação variada de sintomas e comorbidades. Portanto, os percentuais para essas variáveis não somam 100%, pois dependem da quantidade de sintomas e comorbidades referidas pelos pacientes.

SatO_2_ - saturação de oxigênio; UTI - unidade de terapia intensiva.

Com relação ao contexto social das crianças e adolescentes, as notificações se concentraram em indivíduos vivendo na zona urbana (n = 384; 85,5%), residentes em João Pessoa (n = 139; 28,5%) e em municípios de grande porte (n = 268; 55,1%); com densidade demográfica acima de 165,52 habitantes/km^2^ (n = 242; 49,8%); Índice de Desenvolvimento Humano Municipal (IDHM) alto (n = 217; 44,7%); Índice de Vulnerabilidade Social (IVS) baixo (n = 231; 47,5%); taxas de mortalidade infantil entre 10,32% e 12,92% (n = 177; 38,6%); taxa de analfabetismo até os 18 anos de idade compreendendo a faixa de 13,08 até 28,83% (n = 244; 50,2%); cobertura de 23 a 199 estabelecimentos com a Estratégia Saúde da Família (n = 236; 48,5%); de 34 a 208 estabelecimentos de saúde (n = 233; 47,9%); disponibilização de 25 a 249 leitos pediátricos (n = 219; 45,0%); percentual de pavimentação de vias urbanas entre a 17,0% e 25,1% (n = 227; 46,6%); esgotamento sanitário de 56,2 até 70,8% (n = 164; 33,8%) (Tabela 2).

**Tabela 2:** Variáveis contextuais dos pacientes notificados com COVID-19

**Variável**	
Zona (n = 449)	
Urbana	384 (85,5)
Rural	64 (14,3)
Periurbana	1 (0,2)
Município de residência (n = 486)	
João Pessoa	139 (28,6)
Campina Grande	61 (12,6)
Mamanguape	28 (5,8)
Santa Rita	14 (2,9)
Cajazeiras	13 (2,6
Caaporã	12 (2,4)
Patos	10 (2,1)
Bayeux	9 (1,9)
Cabedelo	9 (1,9)
Monteiro	7 (1,4)
Guarabira	6 (1,2)
Cidades com 5 casos	6 (6,2)
Cidades com 4 casos	4 (3,2)
Cidades com 3 casos	9 (5,6)
Cidades com 2 casos	25 (10,3)
Cidades com 1 caso	55 (11,3)
Porte populacional (n = 486)	
Grande porte	268 (55,1)
Médio porte	162 (33,3)
Pequeno porte	56 (11,5)
Densidade demográfica (n = 486), habitantes por km^2^	
Até 88,84	124 (25,5)
88,85 - 165,52	120 (24,7)
165,53 - 4321,28	242 (49,8)
IDHM (n = 486)	
Baixo	173 (35,6)
Médio	96 (19,5)
Alto	217 (44,7)
IVS (n = 486)	
Baixa vulnerabilidade	231 (47,5)
Média vulnerabilidade	47 (9,7)
Alta vulnerabilidade	145 (29,8)
Muito alta vulnerabilidade	63 (13,0)
Taxa de mortalidade infantil (n = 459), Óbitos por 1.000 nascidos vivos	
Até 10,31	115 (25,1)
10,32 - 12,92	177 (38,6)
12,93 - 13,55	65 (14,1)
Acima de 13,55	102 (22,2)
Taxa de analfabetismo (n = 486), %	
13,08 - 28,83	244 (50,2)
28,84 - 40,34	129 (26,5)
Acima de 40,34	113 (23,3)
Cobertura da ESF (n = 486), unidades	
Até 8	137 (28,2)
9 - 22	113 (23,3)
23 - 199	236 (48,5)
Estabelecimentos de saúde (n = 486)	
Até 12	141 (29,0)
13 - 33	112 (23,1)
34 - 208	233 (47,9)
Total de leitos pediátricos (n = 486)	
Até 4	134 (27,5)
5 - 24	133 (27,4)
25 - 249	219 (45,1)
Pavimentação de vias urbanas (n = 484), %	
Até 3,9	136 (28,1)
4,0 - 16,9	109 (22,5)
17,0 - 25,1	227 (46,9)
Acima de 25,1	12 (2,5)
Esgotamento sanitário (n = 485), %	
Até 21,1	127 (26,2)
21,2 - 56,1	118 (24,3)
56,2 - 70,8	164 (24,3)
Acima de 70,8	76 (15,7)
Índice de Gini (n = 486), renda domiciliar per capita	
Valores até 0,5182	122 (25,1)
Valores de 0,5183 até 0,5640	132 (27,2)
Valores de 0,5641 até 0,6290	223 (45,9)
Valores acima de 0,6291	9 (1,8)

ESF - Estratégia Saúde da Família. IDHM - Índice de Desenvolvimento Humano Municipal; IVS – Índice de Vulnerabilidade Social.

Nos modelos estatísticos elaborados, algumas variáveis contínuas apresentaram melhor ajuste. Idade e Índice de Gini tiveram melhor ajuste no modelo de regressão logística como variável contínua. No teste individual da variável explicativa com o desfecho, o Índice de Gini na forma categórica (definidas as categorias pelos quartis) obteve valor de p de 0,332, não sendo considerada para o próximo teste. Já a variável categórica idade (faixas etárias também definidas pelos quartis) obteve valor de p < 0,20 no teste com variável única e o desfecho, entretanto, na elaboração do modelo de regressão ajustado, não apresentou significância estatística (valores de p para idade de zero a 4 anos igual a 1; acima de 4 - 9 anos igual a 0,187; acima de 9 - 15 anos igual a 0,3343; idade acima de 15 - 18 anos igual a 0,111), considerando para o modelo final α de 5%. O porte populacional, apresentou melhor ajuste na forma categórica na regressão logística, e, para a regressão logística multinível, permaneceu no modelo final como variável contínua.

Nas análises bivariadas das regressões logísticas simples, as variáveis que apresentaram valor de p ≤ 0,20 e foram levadas para o modelo de regressão logística múltipla podem ser visualizadas na tabela 3. Os resultados do modelo de regressão logística múltipla final podem ser visualizados na tabela 4. Referente à idade, à medida que esta aumenta em anos reduz em 6% (razão de chance - RC = 0,935; intervalo de confiança - IC95% 0,901 - 0,971) a chance de internamento na UTI. Com relação à variável sexo, crianças e adolescentes do sexo masculino apresentam chance de cuidados intensivos 98% (RC = 1,981; IC95% 1,181 - 3,322) maior que crianças e adolescentes do sexo feminino. A tosse e a febre foram consideradas fatores que reduziram a probabilidade de internamento, indicando que os pacientes pediátricos que apresentavam esses sintomas tinham 68% (RC = 0,322; IC95% 0,175 - 0,593) e 58% (RC = 0,415; IC95% 0,234 - 0,737) menos chances de ir para a UTI, respectivamente. A ocorrência do desconforto respiratório e da dispneia aumentou 2,43 (RC = 2,428; IC95% 1,293 - 4,562) e 3,56 (RC = 3,565; IC95% 1,771 - 7,175) vezes as chances de crianças e adolescentes com indicação de cuidados intensivos em relação aos pacientes que não relataram esses sintomas.

**Tabela 3:** Análise bivariada do internamento de crianças e adolescentes com COVID-19 na unidade de terapia intensiva

**Variável**	**Valor de p**	**RC**	**IC95%**
Sexo			
Feminino	-	1	-
Masculino	0,009	1,709	1,142 - 2,563
Idade (anos)	0,001	0,999	0,9997 - 0,9999
0 - 4	-	1	-
5 - 9	0,022	0,456	0,225 - 0,869
10 - 15	0,007	0,411	0,209 - 0,767
16 - 18	0,008	0,509	0,305 - 0,832
Regional de Saúde da Residência			
I NRS João Pessoa	-	1	-
III NRS Campina Grande	0,134	1,495	0,877 - 2,519
IV NRS Cuite	0,136	6,290	0,593 - 136,718
V NRS Monteiro	0,078	3,145	0,849 - 11,654
XI NRS Princesa Isabel	0,093	4,718	0,765 - 36,430
Porte populacional			
Pequeno porte	-	1	-
Grande porte	0,201	1,589	0,805 - 3,382
Índice de Gini	0,118	0,065	0,002 - 1,989
Tosse			
Não	-	1	-
Sim	0,014	0,592	0,389 - 0,897
Febre			
Não	-	1	-
Sim	0,006	0,548	0,357 - 0,841
Dor de garganta			
Não	-	1	-
Sim	0,069	0,564	0,295 - 1,024
Dispneia			
Não	-	1	-
Sim	2,52e-05	2,533	1,647 - 3,939
Desconforto respiratório			
Não	-	1	-
Sim	4,8e-07	3,148	2,022 - 4,973
Diarreia			
Não	-	1	-
Sim	0,071	0,558	0,286 - 1,026
Saturação < 95%			
Não	-	1	-
Sim	1,14e-06	3,156	1,986 - 5,041
Vômitos			
Não	-	1	-
Sim	0,027	0,472	0,232 - 0,472
Outros sintomas			
Não	-	1	-
Sim	0,0270	0,559	0,331 - 0,928
Presença de algum fator de risco			
Não	-	1	-
Sim	0,016	0,599	0,367 - 0,711
Puérpera			
Não	-	1	-
Sim	0,017	0,214	0,049 - 0,669
Asma			
Não	-	1	-
Sim	0,078	0,252	0,038 - 0,960
Problemas neurológicos			
Não	-	1	-
Sim	0,056	2,333	0,973 - 5,622
Raio X			
Normal	-	1	-
Infiltrado intersticial	0,061	2,431	0,967 - 6,2960
Outras imagens	0,022	3,417	1,197 - 10,027

RC - razão de chances; IC95% - intervalo de confiança de 95%; NRS - Núcleo Regional de Saúde.

**Tabela 4:** Modelo de regressão logística final ajustado dos fatores associados ao internamento por COVID-19 de crianças e adolescentes em unidade de terapia intensiva

**Variável**	**Valor de p**	**RC**	**IC95%**
Intercepto	0,189	5,104	0,446 - 58,345
Idade	0,000	0,935	0,901 - 0,971
Sexo			
Feminino	-	1	-
Masculino	0,009	1,981	1,181 - 3,322
Tosse			
Não	-	1	-
Sim	0,000	0,322	0,175 - 0,593
Febre			
Não	-	1	-
Sim	0,003	0,415	0,234 - 0,737
Desconforto respiratório			
Não	-	1	-
Sim	0,006	2,428	1,293 - 4,562
Dispneia			
Não	-	1	-
Sim	0,000	3,565	1,771 - 7,175
Porte populacional			
Pequeno porte	-	1	-
Grande porte	0,035	2,696	1,074 - 6,767
Índice de Gini	0,010	0,003	0,000 - 0,243

RC - razão de chances; IC95% - intervalo de confiança de 95%.

Quanto ao porte populacional, crianças e adolescentes com COVID-19 residentes em cidades de grande porte tiveram 2,70 (RC = 2,696; IC95% 1,074 - 6,767) vezes mais chances de serem internados na UTI comparados aos pacientes procedentes de municípios de pequeno e médio porte. Já o Índice de Gini pareceu exercer grande influência no desfecho, pois, à medida em que houve incremento no valor desse coeficiente, houve diminuição acentuada de 99% (RC = 0,003; IC95% 0,000 - 0,243) na chance de internamento de crianças e adolescentes com COVID-19 na UTI (Tabela 4).

A curva ROC aplicada indicou bom ajuste do modelo, apresentando área sob a curva de 0,799 ou 79%, valor que foi considerado ponto de corte. A sensibilidade (verdadeiros positivos) correspondeu a 81,4%, e a especificidade (falsos positivos) teve valor de 67,5%.

Para o modelo de regressão multinível, foi obtido o CCI, que teve como valor 0,146, indicando que 15% da chance de internamento na UTI de crianças e adolescentes com COVID-19 na Paraíba foram explicadas pela caracterização do município. Em seguida, foram testadas as variáveis independentes com a variável resposta, e foi obtido o modelo final, considerando α de 0,05, que pode ser observado na tabela 5.

**Tabela 5:** Modelo de regressão logística multinível final ajustado para avaliar a associação dos fatores individuais e contextuais com o desfecho internamento na unidade de terapia intensiva de crianças e adolescentes com COVID-19

**Variável**	**Valor de p**	**RC**	**IC95%**
Intercepto	< 2e - 16	5,104	0,446 - 58,345
Sexo			
Feminino	-	1	-
Masculino	< 2e - 16	1,694	1,680 - 1,707
Raça			
Não pardos	-	1	-
Pardos	2,36e - 16	0,981	0,973 - 0,989
Índice de Gini	0,0493	0,022	0,021 - 0,022
Porte populacional	3,93e - 15	1,019	1,011 - 1,027
Dispneia			
Não	-	1	-
Sim	0,071	0,996	0,988 - 1,004

RC - razão de chances; IC95% - intervalo de confiança de 95%.

Nessa análise multinível, a chance de crianças e adolescentes do sexo masculino de admissão na UTI era 1,69 vez maior (RC = 1,69; IC95% 1,68 - 1,71) comparados aos pacientes do sexo feminino. A variável raça indicou no modelo que àqueles que se consideravam pardos tinham chance 2% menor (RC = 0,98; IC95% 0,97 - 0,99) de precisarem de cuidados intensivos do que os que não se autodeclararam com essa cor. Essa variável não entrou no modelo de regressão não multinível (valor de p > 0,20), pois apresentou os valores de p de 0,96, 0,59, 0,98, 0,98 e 1,00, respectivamente, para as categorias pardos, pretos, amarelos, indígenas e brancos. Além disso, foram observadas as seguintes frequências dos pacientes com relação à raça (n = 430) internados na UTI: pardos, com n = 92/326 (28,2%), e não pardos, com 26/104 (25%). Para o Índice de Gini, à medida que aumenta seu valor, a chance de internamento da UTI diminui 98% (RC = 0,02; IC95% 0,02 - 0,02). O crescimento do porte populacional a cada 100 mil habitantes, aumenta 1,02 vez (RC = 1,02; IC95% 1,01 - 1,03) a chance de indicação de crianças e adolescentes com COVID-19 para os cuidados críticos. A variável dispneia foi importante para o ajuste do modelo, porém não foi significativa a 5%.

O valor da *deviance* obtido com o modelo foi 479,432, indicando bom ajuste. Além da *deviance*, a curva ROC foi realizada, com valor de 0,691 da área sob a curva e respectivo intervalo de confiança (IC95% 0,637 - 0,743), indicando boa qualidade do modelo encontrado.

## DISCUSSÃO

Os modelos estatísticos elaborados expressam que o internamento na UTI de crianças e adolescentes com COVID-19, no estado da Paraíba, esteve associado a variáveis próprias do indivíduo e também ao contexto no qual os pacientes estão inseridos. Esses achados evidenciam o caráter social da doença e reforçam a necessidade de considerar, dentre as variáveis de interesse a serem analisadas, determinantes contextuais que possam influenciar na condição de saúde das crianças e dos adolescentes. Foi observado, ainda, que a utilização de um modelo multinível apresentou diferenças nas estimativas dos parâmetros, em comparação a outros tipos de modelos estatísticos, tornando-se relevante esse tipo de modelagem. Os resultados para o modelo de regressão logística múltipla sem níveis hierárquicos apontam como variáveis que influenciam no internamento de crianças e adolescentes na UTI idade, sexo masculino, tosse, febre, desconforto respiratório, dispneia, porte populacional e Índice de Gini. Outros estudos também utilizaram a regressão logística para avaliar a relação entre variáveis contextuais e o desfecho.^(11,12)^ Já na regressão multinível, sexo, raça, Índice de Gini e porte populacional foram as variáveis que ingressaram no modelo final e influenciaram no desfecho.

A análise revelou que, à medida que aumenta a idade em anos, há redução na chance de esses pacientes necessitarem de cuidados intensivos. Os indivíduos de menor idade parecem ser afetados de forma diferente por quadros clínicos mais graves de COVID-19. Crianças mais jovens apresentaram maior frequência de hospitalização e necessidade de UTI comparadas a crianças maiores.^(13)^ Maior sensibilidade à desidratação e vacinação incompleta são sugeridas como fatores que aumentariam o risco de complicações da COVID-19 em crianças menores de 1 ano, segundo estudo iraniano.^(14)^

Nos dois modelos estatísticos, crianças e adolescentes do sexo masculino apresentam mais chances de precisarem de cuidados intensivos. Alguns trabalhos evidenciam as diferenças da ocorrência da COVID-19 entre os sexos.^(15-17)^ Estudo multicêntrico realizado em 19 UTIs no Brasil mostrou que a maioria dos pacientes internados era do sexo masculino,^(18)^ concordando com esses resultados.

Trabalhos realizados com pacientes adultos apontam que ocorrem diferenças biológicas entre homens e mulheres, como a expressão de enzima conversora de antiotensina (ACE2) e de enzima protease serina transmembranar 2 (TMPRSS2), responsáveis pela entrada do coronavírus da síndrome respiratória aguda grave 2 (SARS-CoV-2) na célula e pela invasão viral, e a regulação por hormônios sexuais exercida nessas estruturas pode sugerir maior letalidade da COVID-19 para a população masculina.^(15,16)^ Acredita-se que essas hipóteses também expliquem o maior acometimento de meninos por quadros mais graves, uma vez que ainda são escassos estudos voltados ao público infantil que revelem essa predominância.

Nos resultados da regressão logística, pacientes pediátricos que apresentavam tosse e febre tinham menor chance de ir para a UTI. Esses sintomas são definidos como alguns dos sinais de alerta nos pacientes com suspeita da COVID-19 segundo a Agência Nacional de Vigilância Sanitária (ANVISA),^(19)^ e o seu aparecimento no início da infecção pode levar à busca pelo atendimento médico precoce, com chances de minimizar o agravamento do quadro.

Os sintomas de desconforto respiratório e dispneia foram considerados fatores que contribuem para a ocorrência do desfecho. Corroborando esses achados, estudos apontam que crianças e adolescentes com sintomatologia de infecção do trato respiratório apresentam maior probabilidade de requerer internação na UTI,^(20)^ sendo a falta de ar significativamente associada à gravidade da COVID-19.^(21)^ A dispneia foi relatada como o achado mais comum nos casos graves em neonatos.^(22)^ A deficiência de vitamina D foi relatada como uma das causas para dispneia em crianças iranianas hospitalizadas que cursaram com quadro clínico grave da COVID-19.^(14)^

Pacientes que se autodeclararam pardos tinham menor probabilidade de serem internados na UTI em relação àqueles que não declararam essa cor. Os achados são contraditórios com a literatura^(23-25)^ inclusive, pois a maioria dos internamentos na UTI foi de pacientes pardos para este estudo. Ao longo do período pandêmico, novas hipóteses surgiram para explicar a relação entre a raça e a COVID-19, como a identificação de tipos sanguíneos com mais chances de infecção pelo SARS-CoV-2 associados a brancos e hispânicos.^(26)^

É possível que o fato de a amostra apresentar majoritariamente indivíduos pardos comparados às outras raças explique a raça/cor parda como fator de proteção para internamento de crianças e adolescentes na UTI. Aqueles que se autodeclararam pardos foram a maioria também entre os que evoluíram para a cura neste trabalho. Isso pode ser reflexo do que a Pesquisa Nacional por Amostra de Domicílios Contínua Trimestral (PNAD Contínua)^(27)^ identificou em seus registros: a população paraibana é predominantemente parda (59,6%). Outra explicação para o resultado encontrado pode estar na estratificação do banco de dados, que separou os indivíduos em pardos, brancos, negros, amarelos e indígenas, não agrupando pretos e pardos no mesmo grupo. Dessa forma, a análise considerou apenas dois grupos: os que não se declararam pardos e os pardos. Além disso, a mineração dos dados em níveis hierárquicos apresentou a influência dos *clusters* (municípios) sobre o desfecho, o que não ocorre no modelo de regressão não multinível que trabalha apenas com um nível, gerando um modelo com algumas variáveis explicativas diferentes do modelo de regressão não multinível, incluindo raça. Portanto, apesar da significância estatística da raça/cor parda, esse resultado precisa ser analisado com cautela, visto que trabalhos^(23-25)^ apontam a repercussão desfavorável da COVID-19 em indivíduos pardos.

Quanto ao porte populacional, nos modelos de regressão logística sem e com multinível, crianças e adolescentes com COVID-19 residentes em cidades de grande porte têm mais chances de serem internados na UTI. O Ceará e o Piauí também apresentaram maiores índices de propagação da doença em suas capitais e em municípios circunvizinhos, fato explicado pela alta densidade populacional, colaborando para a rápida propagação do vírus.^(28,29)^ A disseminação de doenças infecciosas provocadas por vírus tem ligação estreita entre o deslocamento de pessoas, a urbanização e o movimento de estrangeiros, características inerentes às grandes metrópoles.^(30)^ Além disso, municípios acima de 400 mil habitantes apresentam níveis mais elevados de despesas *per capita* com saúde combinado com valores mais altos do repasse do Sistema Único de Sáude (SUS), como também o direcionamento de mais receitas próprias em saúde. Ademais, à medida que cresce a população, esses municípios assumem papéis significativos como referência regional para atender a comunidade, incorporando procedimentos de média e alta complexidade,^(31)^ aumentando consequentemente o número de internações em leitos de cuidados intensivos.

O Índice de Gini aparece nos dois modelos e interfere em uma relação inversamente proporcional, diminuindo as chances de internamento na UTI à medida que seus valores aumentam. À medida que o valor do Índice de Gini aumenta, maior desigualdade na distribuição de renda é observada. Um alto valor no Índice de Gini pode resultar no distanciamento de segmentos populacionais, existindo poucas chances de integração social e, dessa forma, de interromper inicialmente o aumento de transmissão do SARS-CoV-2, diminuindo a disseminação do vírus.^(32)^

A utilização de dois métodos estatísticos com e sem níveis hierárquicos para modelagem dos dados reforçou a relação das variáveis preditoras obtidas nos modelos finais influenciando o mesmo desfecho. A regressão logística sem níveis hierárquicos apresenta a repercussão de variáveis explicativas sobre o internamento de crianças e adolescentes com COVID-19 na UTI de maneira mais generalizada, sem levar em consideração as características que podem existir no agrupamento por município. Já o modelo multinível permite a exploração dos dados com mais detalhes, evidenciando a variabilidade entre os níveis com relação ao desfecho. Nesse sentido, a incorporação dos efeitos aleatórios dos grupos, no caso dos municípios, foi relevante para estimação dos parâmetros, quando as respostas estão agrupadas.

Ressalta-se que os dois modelos estatísticos trazem informações importantes acerca do evento de interesse, e analisar a contribuição de diversos resultados apresentados por modelagens diferentes pode favorecer o entendimento de como crianças e adolescentes com COVID-19 evoluiriam para o internamento na UTI, considerando aspectos mais abrangentes e características comuns intrínsecas a determinados agrupamentos, favorecendo o desenvolvimento de estratégias mais assertivas.

Vale salientar que essas informações devem ser interpretadas com cautela, uma vez que se trata de dados secundários, com risco de subnotificação. Além disso, as dificuldades de acesso aos serviços de saúde e testagem da população, especialmente das crianças e dos adolescentes, podem subestimar o real número de casos da doença, tornando-se limitações deste estudo. Outra limitação está relacionada às variáveis coletadas em bancos de dados públicos, como o Departamento de Informática do SUS (DATASUS) e o Atlas da Vulnerabilidade Social, que não têm atualização recente, prejudicando a comparação com as informações atuais, visto que muitas crianças e adolescentes nasceram anos depois da publicação desses dados. Apesar das limitações apresentadas, as informações geradas neste estudo apontam a necessidade de considerar variáveis contextuais para uma melhor compreensão do curso da COVID-19 em crianças e adolescentes com COVID-19 que necessitam de cuidados intensivos.

## CONCLUSÃO

Os resultados sugerem que a associação entre condições próprias do paciente e o agravamento da infecção pelo SARS-CoV-2, resultando na necessidade de cuidados intensivos, pode ser influenciada pelo contexto social e econômico em que crianças e adolescentes estão inseridos, e a magnitude desses efeitos é melhor estimada com a inclusão de um modelo de regressão multinível nas análises.

Destarte as considerações feitas, é possível sugerir que os perfis clínico e socioeconômico da população possam direcionar o desenvolvimento de políticas no combate ao coronavírus, tornando-se necessário olhar cautelosamente o público infantojuvenil, que vem crescendo no número de casos e mortes ao longo do tempo, em meio a um panorama pandêmico muito instável, com o aparecimento de novas variantes e a restrição de vacinas que atendam a todas as crianças, especialmente as mais jovens - faixa etária especialmente afetada. Conduzir estratégias pelo prisma da realidade clínica e social provavelmente é mais assertivo no controle e na mitigação do SARS-CoV-2 nessa população.
